# Comparative cost of illness analysis and assessment of health care burden of Duchenne and Becker muscular dystrophies in Germany

**DOI:** 10.1186/s13023-014-0210-9

**Published:** 2014-12-18

**Authors:** Olivia Schreiber-Katz, Constanze Klug, Simone Thiele, Elisabeth Schorling, Janet Zowe, Peter Reilich, Klaus H Nagels, Maggie C Walter

**Affiliations:** Friedrich-Baur-Institute, Department of Neurology, Ludwig-Maximilians-University of Munich, Ziemssenstrasse 1, 80336 Munich, Germany; Institute for Healthcare Management and Health Sciences, University of Bayreuth, Prieserstrasse 2, 95444 Bayreuth, Germany

**Keywords:** Duchenne muscular dystrophy, Becker muscular dystrophy, Direct costs, Indirect costs, Informal care costs, Cost of illness (COI), Burden of illness, Health care burden, Health-related quality of life (HRQOL), Socio-economic evaluation

## Abstract

**Background:**

Our study aimed to determine the burden of illness in dystrophinopathy type Duchenne (DMD) and Becker (BMD), both leading to progressive disability, reduced working capacity and high health care utilization.

**Methods:**

A micro-costing method was used to examine the direct, indirect and informal care costs measuring the economic burden of DMD in comparison to BMD on patients, relatives, payers and society in Germany and to determine the health care burden of these diseases. Standardized questionnaires were developed based on predefined structured interview guidelines to obtain data directly from patients and caregivers using the German dystrophinopathy patient registry. The health-related quality of life (HRQOL) was analyzed using PedsQL™ Measurement Model.

**Results:**

In total, 363 patients with genetically confirmed dystrophinopathies were enrolled. Estimated annual disease burden including direct medical/non-medical, indirect and informal care costs of DMD added up to € 78,913 while total costs in BMD were € 39,060. Informal care costs, indirect costs caused by loss of productivity and absenteeism of patients and caregivers as well as medical costs of rehabilitation services and medical aids were identified as the most important cost drivers. Total costs notably increased with disease progression and were consistent with the clinical severity; however, patients’ HRQOL declined with disease progression.

**Conclusion:**

In conclusion, early assessments of economic aspects and the disease burden are essential to gain extensive knowledge of a distinct disease and above all play an important role in funding drug development programs for rare diseases. Therefore, our results may help to accelerate payer negotiations such as the pricing and reimbursement of new therapies, and will hopefully contribute to facilitating the efficient translation of innovations from clinical research over marketing authorization to patient access to a causative treatment.

**Electronic supplementary material:**

The online version of this article (doi:10.1186/s13023-014-0210-9) contains supplementary material, which is available to authorized users.

## Background

Duchenne muscular dystrophy (DMD) is a hereditary X-linked neuromuscular disorder due to mutations in the dystrophin gene with a worldwide incidence of approximately 1:5,000 male newborns [1,2] leading to progressive muscle atrophy and weakness. First symptoms are usually noted between the age of three to five, while loss of ambulation occurs around age 12, along with scoliosis, contractures, respiratory and cardiac impairment which require early ventilatory support and drug treatment. Life expectancy is reduced to 30-40 years of age although multidisciplinary symptomatic and surgical treatment has considerably improved survival within the last two decades [3-5].

Becker muscular dystrophy (BMD) represents a milder allelic form of dystrophinopathy, with a broader variability of clinical symptoms, a later onset, milder impairment and slower progression with an estimated incidence of 1:20,000 [6,7]. However, only symptomatic treatment is available for slowing down disease progression in both forms of the disease. Therefore, Duchenne and Becker muscular dystrophy inevitably result in reduced working capacity and costly health care utilization for patients and caregivers. Until now, the burden of DMD and BMD regarding patients, family caregivers, health care systems and society has scarcely been investigated [8]. Our study aimed to assess the cost of illness (COI) and health-related quality of life (HRQOL) of both DMD and BMD patients in Germany, to forecast and model the burden of illness from a health economic and clinical perspective.

## Methods

We performed a cross-sectional study approaching all male patients with a confirmed genetic diagnosis of DMD or BMD (n = 733) via the German dystrophinopathy patient registry (www.dmd-register.de) and/or their caring relatives (hereafter referred to as “parents”), respectively, to evaluate the burden of dystrophinopathy. The dystrophinopathy patient registry was established within the network of excellence TREAT-NMD (www.treat-nmd.eu) in 2008 as described elsewhere [9]. Standardized questionnaires adapted to either DMD or BMD were developed in close cooperation with clinicians, economists and representatives of German patient organizations, consisting of several sections addressing patients’ and parents’ socio-demographic data, health status, use of resources over a specific time period and the current or previous employment of patients and parents. The PedsQL™ Measurement Model, module for neuromuscular disorders, German version 3.0, was applied to analyze the quality of life. Following a pretesting phase with personal interviews with patients and caregivers, the survey started in June 2013 and was closed in December 2013. Questionnaires were sent out to patients and parents either by mail or online and were answered either by adolescent/adult patients aged 16 and older or by their parents (in the case of younger patients or patients dependent on part- or fulltime care). Based on the subjective evaluation of patients/parents, DMD and BMD patients were classified into disease severity stages mirroring disease progression and motor function (Table 1). Thereby the differences between DMD and BMD, and within their disease progression could be analyzed. Ethical approval was obtained from the ethics board of the Ludwig-Maximilians-University of Munich; and patients/legal guardians and parents gave their written consent to participate in the study.

**Table 1:** **Definition of patient**/**parent self**-**evaluated clinical severity stages**

**Stage**	**Clinical characteristics of DMD and BMD boys**/**men**
I	*Early ambulatory with mild impairment*: Gowers’ maneuver, waddling gait, walking on toes, problems with climbing stairs.
II	*Late ambulatory with high impairment*: Walking becomes increasingly difficult, more problems climbing stairs and getting up from the floor, part-time wheelchair use.
III	*Early non-ambulatory*: Loss of ambulation, active manual wheelchair use possible, independent standing and sitting still possible for some time.
IV	*Late non-ambulatory*: Independent electric wheelchair use but decline of upper limb function and ability to sit independently.
V	*Non-ambulatory with confinement to bed*: Loss of independent mobility, hand function preserved on a low level.

According to self-evaluated clinical symptoms and motor function DMD and BMD patients were divided into corresponding subgroups to reveal potential correlations between these functional stages and the burden of illness. *Modification after Bushby K et al*., *The Lancet Neurology*, *2010* [3].

A micro-costing method was used to retrospectively examine the direct, indirect and informal care costs from a societal perspective. To evaluate the direct COI - including e.g. costs of hospitalization, drug treatment, rehabilitation services such as physiotherapy and occupational therapy - the use of resources was identified and monetarily assessed using the official German price lists of 2013 [10,11]. Incurred costs of health care services were extrapolated to one year, assuming constant use of resources. Cost drivers as constructional adaptions of the environment as well as advocate support were estimated as cumulative per-patient mean costs that incurred up to the date the questionnaire was completed. In addition the costs of medical aids and respiratory management were estimated as stage-specific mean costs. By extrapolating the mean duration of DMD and BMD stages within our patient cohort based on age at diagnosis and age and disease stage at the time of the study participation, the annual costs were estimated for these cost categories, respectively. For assessing costs of informal care, only the care effort of non-working parents was calculated in hours per day preventing an overestimation of indirect and informal care costs by double counting working parents’ care time and their loss of productivity. We developed a formula to calculate the economic loss of productivity caused by absenteeism, invalidity or changes in the work situation of patients and parents by analyzing patients and parents indirect COI (see Additional file 1). Compared to the human capital approach, the developed formula delivers a more precise description of the real-life situation by taking factors such as short-time absenteeism or the actual wage levels into account [12]. Differences in costs and HRQOL between DMD and BMD were analyzed using the Mann-Whitney-U-test within the SPSS® software package; results are presented in Euro for 2013.

## Results

### Patient cohort characterization

363 patient/parent pairs were included in the analysis (response rate 50%: 363/733; DMD 43% = 248/571, BMD 71% = 115/162). Most DMD respondents represented clinical severity stages IV, II and I in contrast to the majority of BMD patients who were in stages II and I (none was in stage V). The age of the DMD patients ranged from 1 to 42 years (median 11y), while BMD patients were between 2.5 and 62 years old (median 26.5y); however, median age increased with clinical severity (except for BMD stage III; Table 2).

**Table 2:** **Response rate and age distribution within the analyzed patient cohort**

	**DMD**						**BMD**					
	**Total**	**I**	**II**	**III**	**IV**	**V**	**Total**	**I**	**II**	**III**	**IV**	**V**
**Response rate**												
Absolute numbers	248/571	49	70	11	92	26	115/162	42	55	8	10	0
Ratio of total DMD/BMD [%]	43	20	28	4	37	11	71	37	48	7	9	0
Respondents patients/parents [%]	10/90						68/32					
**Age of patients**												
Min. [years]	1	1	3	10	1	11	2.5	2.5	7	7	21	-
Max. [years]	42	14	42	23	31	40	62	52	59	41	62	-
Median [years]	11	4	7.5	13	16	22.5	26.5	16	33	30	51	-

Response rates and age distribution were differentiated based on clinical severity stages. Because of rounding, percentage might not add up to exactly 100%.

The assessment of the family status of the patients revealed that most DMD patients (96%) were unmarried whereas in the BMD population nearly 50% were married or in a partnership. The percentage of DMD and BMD parents who were married/in a partnership were 87% and 74%, respectively (Table 3). Health costs were covered by a statutory health insurance in the majority of patients (DMD 91%; BMD 87%), only 9%/13% of DMD/BMD patients had private health insurance (data not shown), which is in line with the ratio within the German population [13]. The German health care system provides long-term nursing care insurance with three different levels of financial support, depending on the patients’ impairment and their need of nursing services [14]. Within the analyzed DMD cohort, 30% of patients were not yet classified into care levels. In contrast, 17% of patients were classified into care level 1 (median age 8y), 23% into care level 2 (median age 13y) and 31% into the most severe care level 3 (median age 20y). Within the BMD population, 76% of patients did not qualify for any of the care levels, 14% were in care level 1 (median age 35.5y) and 4%/5% were classified into care levels 2 and 3 (median age 41/50y; data not shown).

**Table 3:** **Family status**, **education and employment status within the analyzed patient cohort**

		**DMD**		**BMD**	
		**Patients**	**Parents**	**Patients**	**Parents**
**Family status** **[%]**	Widowed	0	2	0	0
	Divorced	0	5	4	9
	Married	4	83	36	71
	In a partnership	0	4	13	3
	Unmarried	96	6	47	17
**Education** **[%]**	No qualification	20	2	0	3
	School	74	78	82	82
	University studies	6	20	18	15
	Completed professional education	39	89	77	82
**Employment status** **[%]**	**Non**-**working**	49	8	32	18
	**Currently working**				
	Self-employed	2	7	8	11
	Employed	37	56	49	67
	**Quit working life**	12	29	12	4
	In stage I	-	17	0	0
	In stage II	0	52	63	50
	In stage III	25	15	13	0
	In stage IV	50	13	25	50
	In stage V	25	3	-	-
	**Reduced working time** **[%]**	6	38	25	12
	In stage I	-	10	22	71
	In stage II	11	55	67	14
	In stage III	11	13	11	14
	In stage IV	67	19	0	0
	In stage V	11	3	-	-
**Gross salary per year** **[€]**	Mean	11,405	24,168	35,871	27,414
	Stage I	-	25,971	45,120	29,937
	Stage II	22,200	25,140	33,377	22,071
	Stage III	2,400	30,722	33,720	40,200
	Stage IV	13,572	22,976	32,644	28,054
	Stage V	6,712	17,745	-	-

These demographic parameters were assessed to serve as basis for indirect cost calculation. Assessment was differentiated between patients and parents in consideration of patients’ clinical severity stages. Because of rounding, percentage might not add up to exactly 100%.

### Estimation of direct medical cost of illness

**Figure 1:**
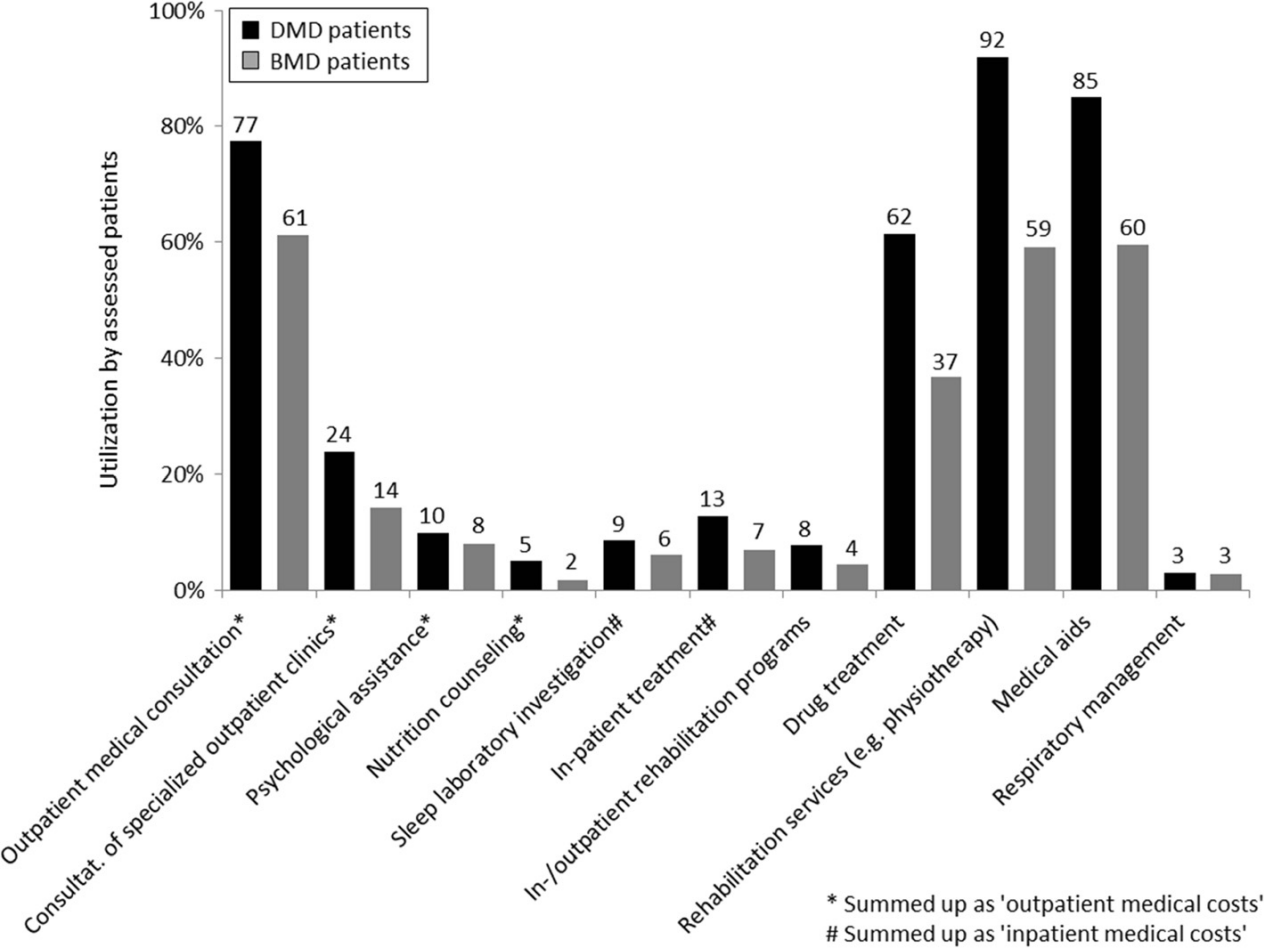
**Consumption of resources of direct medical services by dystrophinopathy patients.** DMD patients showed higher utilization of direct medical services compared to BMD patients (percentage rounded).

The consumption of direct medical resources was notably higher in DMD than in BMD patients. The most striking differences were seen regarding the demand of rehabilitation services (e.g. physiotherapy, occupational therapy and logopedics), drug treatment, use of medical aids and in- and outpatient medical consultations. Additionally, DMD patients required health care resources much earlier than BMD patients; e.g. DMD patients needed in-patient treatments within a median average age of 14 years, while BMD patients were already 40 years of age. Moreover, osteoporosis was more frequently seen in DMD than in BMD, occurring earlier in DMD and requiring medical treatment (12% of DMD patients, median age 20 y, 39% with drug treatment; vs. 3% of BMD patients, median age 44y, none with drug treatment; data not shown). More than three quarters of DMD (84.9%) and more than half of BMD patients (59.6%) stated that they need medical aids. Within the investigated patient cohort, DMD boys became full-time wheelchair bound at a median age of 15 years compared to BMD at a median age of 40 years. In contrast, an assessment of the ongoing need for respiratory management showed that 3% of DMD (each 50% in stages IV and V; aged 2 to 25 y, median 21y) and 2.7% of BMD patients (67%/33% in stage II/IV; aged 7 to 59 y, median 40 y) used an invasive or non-invasive ventilatory device. Overall, DMD patients exhibited a more severe morbidity along with a higher need for direct medical care (Figure 1).

Based on these differences, the mean direct annual COI of DMD was estimated at € 19,346 and thus 3.8 times higher compared to € 5,140 in BMD (Tables 4 and 5). Main direct medical cost drivers were especially costs of medical aids in DMD (13% of total COI), costs of rehabilitation services (DMD/BMD 6% of total COI) and costs of inpatient medical treatment in BMD (3% of total COI). A detailed comparison of direct medical costs between DMD and BMD revealed different patterns of evolving costs through the disease stages, especially a decline of outpatient medical costs and costs of rehabilitation programs from the time-point at which the patients lost their ability to walk, while inpatient medical costs, drug treatment costs, costs of medical aids and respiratory therapy jumped up with disease progression. DMD costs of respiratory management increased to € 2,771 in stage V (mean: € 875), compared to € 234 in BMD stage IV (mean € 83). The costs of medical aids for DMD patients (€ 10,209) were significantly higher compared to BMD (€ 742) and spurted upwards with disease severity (€ 51,019 in stage V), whereas BMD mean costs of medical aids peaked out in stage III on a considerably lower level (€ 3,611). Major cost drivers were aids supporting mobility such as wheelchairs, representing not only the most frequently used aids but also the most expensive devices (as well as other home care tools).

**Table 4:** **Mean annual burden of illness of Duchenne muscular dystrophy in** € (**min**-**max**)

	**Mean**	**Ratio of total COI** **[%]**	**Clinical severity stage**				
			**I**	**II**	**III**	**IV**	**V**
Outpatient medical costs	457 (0-4,644)	1%	279 (0-3,046)	393 (0-4,644)	701 (0-2,973)	552 (0-4,356)	523 (0-2,495)
Inpatient medical costs	1,613 (0-131,454)	2%	585 (0-18,736)	804 (0-20,188)	-	1,540 (0-35,525)	6,673 (0-131,454)
Rehabilitation program costs (in-/outpatient)	1,130 (0-30,053)	1%	-	427 (0-17,017)	2,780 (0-15,290)	2,086 (0-30,053)	954 (0-15,290)
Drug treatment costs	330 (0-5,530)	<1%	172 (0-967)	373 (0-3,837)	344 (0-746)	319 (0-5,530)	550 (0-5,122)
Costs of rehabilitation services (e.g. physiotherapy)	4,732 (0-31,974)	6%	2,693 (0-10,658)	4,042 (0-31,974)	5,204 (0-18,097)	5,817 (0-24,657)	6,478 (0-26,839)
Costs of medical aids	10,209 (0-103,977)	13%	488 (0-4,368)	1,584 (0-11,450)	2,474 (1,285-4,805)	11,153 (0-82,673)	51,019 (1,342-103,977)
Costs of respiratory management	875 (0-12,087)	1%	3 (0-34)	6 (0-34)	163 (0-1,791)	1,522 (0-12,087)	2,771 (0-6,779)
**Total direct medical costs**	**19,** **346**	**25%**	**4,** **220**	**7,** **629**	**11,** **666**	**22,** **989**	**68,** **968**
Costs for the housing situation	4,043 (0-238,800)	5%	55 (0-1,536)	83 (0-2,280)	1,064 (0-6,900)	5,860 (0-192,000)	17,112 (0-238,800)
Costs for personal assistance for school and work attendance	883 (0-28,800)	1%	-	931 (0-15,000)	1,980 (0-9,600)	1,481 (0-28,800)	-
Travel expenses	1,102 (0-17,376)	1%	358 (0-1,479)	741 (0-5,434)	1,814 (108-7,264)	1,714 (0-17,376)	915 (0-3,950)
Advocate support costs	27 (0-8,333)	<1%	1 (0-33)	118 (0-8,333)	0 (0-89)	0 (0-1,623)	0 (0-59)
Investments in house adaptions	3,059 (0-93,636)	4%	2,826 (0-66,667)	0 (0-30,507)	2,239 (0-12,421)	8,499 (0-93,636)	0 (0-13,853)
Investments in automobile adaptions	408 (0-16,697)	1%	62 (0-2,833)	559 (0-11,605)	2,609 (0-12,028)	776 (0-16,697)	0 (0-6,941)
Other expenditures (e.g. artificial nutrition, alternative therapies)	83 (0-12,000)	<1%	41 (0-1,536)	223 (0-12,000)	-	14 (0-735)	510 (0-6,720)
Informal care costs	21,279 (0-223,380)	27%	8,303 (0-77,563)	8,029 (0-62,050)	19,532 (3,103-43,435)	31,490 (0-223,380)	44,443 (0-158,848)
**Total direct non**-**medical costs**	**30,** **884**	**39%**	**11,** **646**	**10,** **684**	**29,** **238**	**49,** **834**	**62,** **980**
**Total direct COI**	**50,** **230**	**64%**	**15,** **866**	**18,** **313**	**40,** **904**	**72,** **823**	**131,** **948**
Indirect costs caused by patients	21,463 (0-43,740)	27%	-	11,100 (11,100-11,100)	-	18,734 (0-43,740)	28,529 (881-43,740)
Indirect costs caused by parents	7,220 (0-324,000)	9%	13,078 (0-324,000)	3,855 (0-24,000)	8,046 (0-18,000)	7,044 (0-45,100)	4,378 (0-12,000)
**Total indirect COI**	**28,** **683**	**36%**	**13,** **078**	**14,** **955**	**8,** **046**	**25,** **778**	**32,** **907**
**Total COI**	**78,** **913**		**28,** **944**	**33,** **268**	**48,** **950**	**98,** **601**	**164,** **855**

Total burden of illness of DMD consisting of total direct medical and non-medical costs and indirect costs was calculated per-patient in € per year while taking disease progression into account. Because of rounding, percentage might not add up to exactly 100%.

**Table 5:** **Mean annual burden of illness of Becker muscular dystrophy in** € (**min**-**max**)

	**Mean**	**Ratio of total COI** **[%]**	**Clinical severity stage**				
			**I**	**II**	**III**	**IV**	**V**
Outpatient medical costs	366 (0-5,618)	1%	438 (0-5,618)	365 (0-2,901)	89 (0-256)	290 (0-1,066)	-
Inpatient medical costs	1,186 (0-56,053)	3%	256 (0-10,767)	678 (0-18,169)	-	8,837 (0-56,053)	-
Rehabilitation program costs (in-/outpatient)	375 (0-22,711)	1%	414 (0-16,977)	471 (0-22,711)	-	-	-
Drug treatment costs	139 (0-2,452)	<1%	55 (0-835)	112 (0-1-162)	286 (0-1,788)	519 (0-2,452)	-
Costs of rehabilitation services (e.g. physiotherapy)	2,249 (0-30,276)	6%	1,252 (0-9,332)	1,988 (0-23,405)	2,404 (0-14,643)	7,744 (0-30,276)	-
Costs of medical aids	742 (0-17,453)	2%	55 (0-894)	658 (0-8,571)	3,611 (0-17,453)	1,725 (164-3,098)	-
Costs of respiratory management	83 (0-1,281)	<1%	0 (0-15)	132 (0-1,281)	5 (0-38)	234 (0-779)	-
**Total direct medical costs**	**5,** **140**	**13** **%**	**2**,**470**	**4,** **404**	**6,** **395**	**19,** **349**	-
Costs for the housing situation	2,182 (0-126,000)	6%	190 (0-7,200)	161 (0-5,400)	300 (0-1,920)	23,933 (0-126,000)	-
Costs for personal assistance for school and work attendance	-	-	-	-	-	-	-
Travel expenses	1,034 (0-14,432)	3%	722 (19-3,752)	925 (0-14,432)	1,539 (0-3,621)	2,215 (377-8,144)	-
Advocate support costs	1 (0-50)	<1%	1 (0-40)	0 (0-27)	-	7 (0-50)	-
Investments in house adaptions	870 (0-26,750)	2%	192 (0-3,000)	1,118 (0-12,583)	4,369 (0-26,750)	0 (0-3,929)	-
Investments in automobile adaptions	719 (0-42,210	2%	-	482 (0-5,000)	7,626 (0-42,210)	0 (0-3,229)	-
Other expenditures (e.g. artificial nutrition, alternative therapies)	29 (0-2,400)	<1%	21 (0-888)	44 (0-2,400)	-	-	-
Informal care costs	7,636 (0-148,920)	20%	5,523 (0-77,562)	4,643 (0-31,025)	3,435 (0-10,859)	33,867 (0-148,920)	-
**Total direct non**-**medical costs**	**12,** **471**	**32%**	**6**,**649**	**7,** **373**	**17,** **269**	**60,** **022**	-
**Total direct COI**	**17,** **611**	**45%**	**9**,**119**	**11,** **777**	**23,** **664**	**79,** **371**	-
Indirect costs caused by patients	18,922 (0-67,014)	48%	18,397 (0-52,756)	18,147 (0-44,394)	7,692 (0-40,500)	29,108 (0-67,014)	-
Indirect costs caused by parents	2,527 (0-33,039)	6%	2,767 (0-33,039)	2,415 0-18,173)	2,105 (0-4,210)	-	-
**Total indirect COI**	**21,** **449**	**55%**	**21**,**164**	**20,** **562**	**9,** **797**	**29,** **108**	-
**Total COI**	**39,** **060**		**30**,**283**	**32,** **339**	**33,** **461**	**108,** **479**	-

Total burden of illness of BMD consisting of total direct medical and non-medical costs and indirect costs was calculated per-patient in € per year while taking disease progression into account. Because of rounding, percentage might not add up to exactly 100%.

### Estimation of direct non-medical cost of illness

In contrast to direct medical costs, total direct costs additionally take non-medical cost factors into account. Both, direct medical and non-medical COI, increased with disease severity with a leap between DMD stage IV and V and BMD stage III and IV. Considerably lower costs of BMD compared to DMD patients were mainly seen in stages I to III. Regarding direct non-medical costs, main cost drivers - apart from informal care costs - were the costs for the housing situation, e.g. caused by a mobile nursing service. These costs were twice as high in DMD as compared to BMD; however, there was a notable escalation with disease progression, resulting in higher mean costs of BMD patients in stage IV compared to DMD patients in stage V. The second important cost driver of non-medical direct costs were expenses due to the progressing physical impairment, e.g. constructional adaptions of the house (DMD 4%; BMD 2% of total COI) and the car (DMD: 1%; BMD 2% of total COI). House adaption costs of both DMD and BMD peaked in the second last stage (DMD € 8,499; BMD € 4,369). Additionally expenses for car adaptions peaked in stage III in DMD and BMD; however, BMD costs were nearly three times as high compared to DMD (DMD € 2,609; BMD € 7,626).

Moreover, informal care costs turned out to be the most important cost driver of direct resource consumption, mainly in severe dystrophinopathies, and also had a notable influence on total health care burden. Informal care provided by family members and friends was assessed as “non-working caregivers’ time providing medical services”, thus representing a loss of leisure time. These costs were counted as the real costs of care by weighing the amount of care with the usual payment of formal care; thereby, informal care costs were attributed to the direct costs [12]. Within the analyzed patient cohort, 89% of DMD and 47% of BMD patients received part- or fulltime care by another person. Parents provided the major part of total care time in DMD (mean 93%). In contrast, in BMD care time was split among parents (51%) and the patients’ partners (41%). Furthermore, siblings assisted in 4%/5% (DMD/BMD) and other relatives in 3%/11% (DMD/BMD) of total care time. Thus, 91% of DMD patients lived together with their parents/families compared to 65% of BMD patients. DMD relatives’ total care efforts in hours per day were higher than those of BMD relatives (mean h/d DMD 9.4, SD: 10.9; mean h/d BMD 2.7, SD: 6.5) with a notable increase in more severe clinical stages. Besides the mere care time, patients needed further support by caregivers, parents and relatives for organizational tasks, mean h/d 1.41 in DMD, and h/d 0.25 in BMD (data not shown).

The mean annual costs of informal care and organizational activities were nearly three times higher in DMD than in BMD (DMD € 21,279; BMD € 7,636; Tables 4 and 5). Costs of care for DMD patients increased with the severity of the clinical phenotype, whereas costs in BMD decreased from stage I to III and peaked in stage IV. When adding care efforts of working parents on top, total informal care costs were estimated even higher (€ 33,402/year in DMD, € 9,224/year in BMD).

Total mean annual non-medical direct costs added up to € 30,884 in DMD and to € 12,471 in BMD. Adding total medical and non-medical costs, total direct mean annual COI of DMD was estimated at € 50,230 and significantly higher than in BMD (€ 17,611; p < 0.001; Tables 4 and 5). In summary, our results show clear-cut differences between the consumption of resources of direct medical and non-medical services and emerging costs not only between DMD and BMD patients, but also between the different stages within both diseases, showing higher costs along with disease progression.

### Estimation of indirect costs caused by patients and parents

The indirect costs in DMD and BMD primarily originate from the loss of working capacity due to the disease, and result in lost working time and overall loss of productivity. Our study differentiated between the lost working time of the patient (see section a) and of his parents (or caregivers) who often had to quit or reduce their employment due to their son’s disease (see section b).

### a) Estimation of indirect costs caused by patients

Within the BMD cohort, the number of patients with access to higher education, mainly university studies and completed professional education, was considerably higher when compared to DMD. The percentage of non-workers was higher in DMD than in BMD (49% vs. 32%) whereas the percentage of patients who had quit their employment in the past was the same in DMD and BMD (12%). Actively working DMD patients (employed or self-employed) were between 18 and 42 years old, whereas working BMD patients ranged from 19 and 59 years of age. DMD patients stopped working or reduced working hours only when they reached more severe clinical stages (50% in stage IV), while 63% of BMD patients already had a cut-off in stage II. DMD and BMD patients reduced working time by mean 16.6 hours/week. The majority of patients (BMD 69%; DMD 82%) felt handicapped in their career, leading to lower income due to physical impairment as stated by 8% of DMD and 35% of BMD patients (data not shown). Overall, DMD patients’ salaries were notably lower than those of BMD patients, in line with the discrepancy regarding the educational level of DMD and BMD patients (DMD: € 11,405; BMD: € 35,871 per year; Table 3). Based on this data, total annual indirect COI of DMD was estimated at € 21,463 vs. € 18,922 in BMD; in both conditions, the total annual indirect COI was highest in the last stage of the disease (BMD stage IV, DMD stage V) with a brief decline in stage III (Tables 4 and 5).

### b) Estimation of indirect costs caused by parents

Nearly two third of the DMD parents were employed or self-employed (BMD 78%). 29% quit their employment to be able to care for their son. 38% of actively working parents reduced their working hours by mean 15 hours/week. In contrast, only 4% of BMD parents quit their job and 12% of actively working parents reduced working time by mean 10 hours/week. DMD parents quit or reduced their employment mainly when their sons reached stage II; most BMD patients were in stage II/IV when their parents stopped working. Moreover, in our study population, parents missed a mean of 14.5 working days per year due to their son’s disease. This is in line with the finding that many parents feel limited in pursuing their career (DMD: 60%; BMD: 17%) and stated to earn less due to their son’s disease (DMD 49% vs. BMD 29%; data not shown). Parents’ gross salaries in 2013 were similar in both diseases (DMD: € 24,168; BMD: € 27,414) and decreased during the progression of the disease (Table 3). Parents’ indirect COI due to absenteeism and reduction of working time was estimated at € 7,220 for DMD vs. € 2,527 for BMD in 2013 (p < 0.01; Tables 4 and 5).

**Figure 2:**
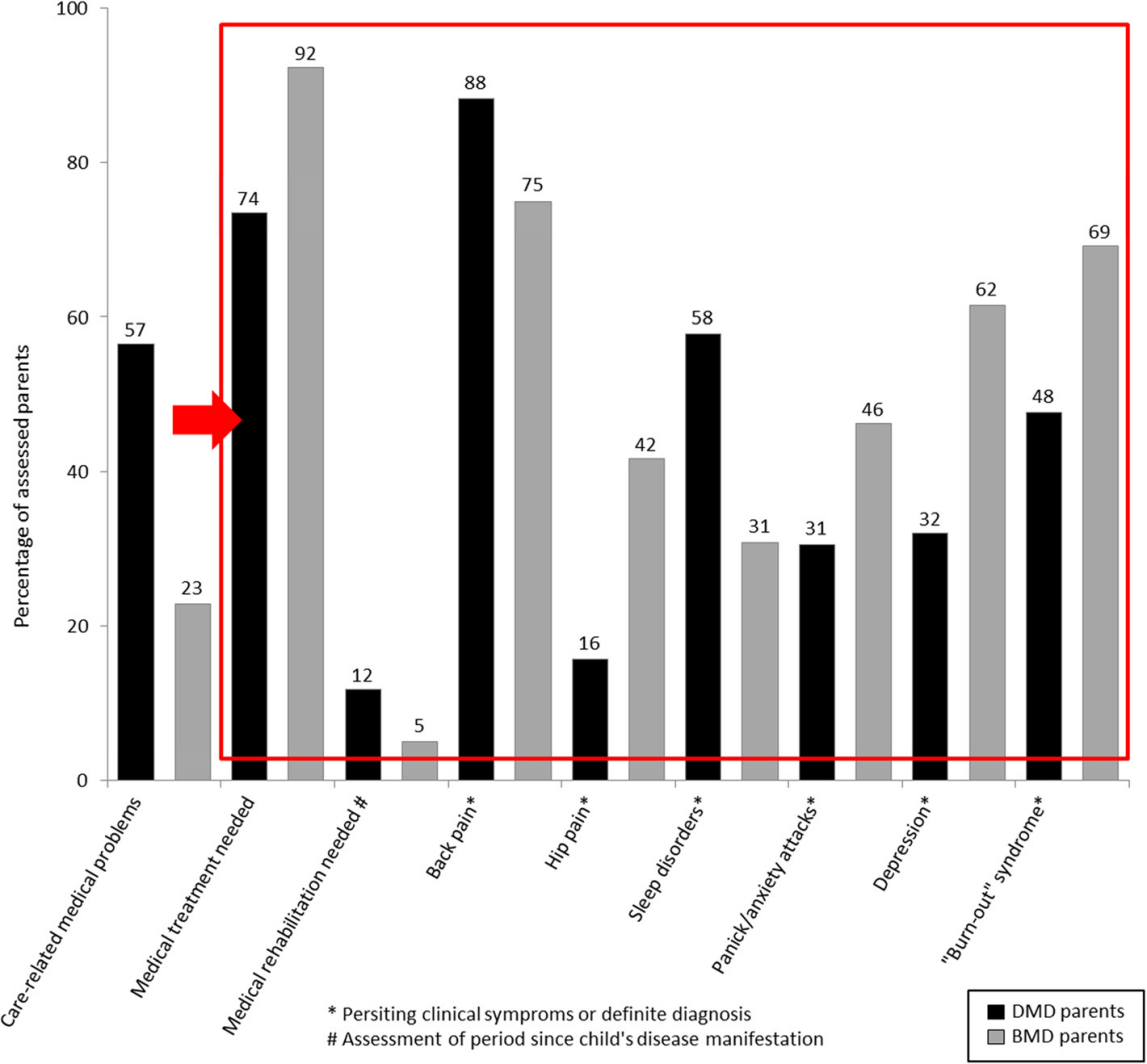
**Subjectively care**-**related medical problems of parents.** More than half of the DMD parents and nearly one quarter of the BMD parents stated to have developed own medical problems related to their son’s disease, leading to consumption of medical treatment and participation in rehabilitation programs (percentage rounded).

In addition to a loss of working capacity in DMD and BMD parents, more than half of the DMD parents and 23% of the BMD parents themselves developed medical problems due to the burden of their son’s disease, leading to further consumption of medical treatment due to parents’ physical or mental problems (Figure 2). Overall, physical and mental problems of parents and caregivers increased with the severity of their son’s impairment. Parents’ own health status not only had an important impact on their ability to care for their son, but also on their working capacity and hence resulting indirect costs.

### Assessment of health-related quality of life

**Figure 3:**
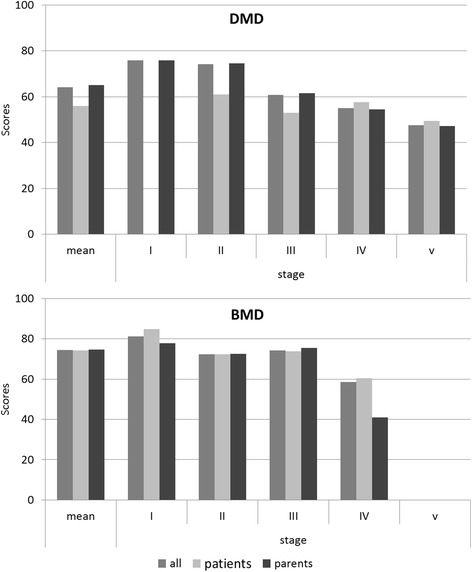
**Health-** **related quality of life of dystrophinopathy patients** **(self** **- or parent-** **reported).** In general, HRQOL of BMD patients was estimated better than that of DMD patients whereas HRQOL declined with disease progression, both in DMD and BMD. HRQOL assessment was performed using PedsQL™ Measurement Model, module for neuromuscular disorders, German version 3.0. Higher scores indicate better HRQOL.

The patients were encouraged to complete the questionnaire by themselves; alternatively, their parents assessed the HRQOL questionnaire. HRQOL results in BMD were better than in DMD (p < 0.001) without relevant differences between the evaluation by patients or parents (p > 0.05). However, DMD patients assessed their HRQOL worse than their parents did (p < 0.05; Figure 3), especially those patients in less advanced clinical severity stages. In general, HRQOL decreased with disease progression with the most prominent loss between stage II and III in DMD and a small increase between stage II and III in BMD. Additionally, patients’ and parents’ contentment with their care/their son’s care was analyzed. The majority of patients were satisfied/very satisfied with their standard of care (DMD 77%; BMD 73%). Parents’ evaluation of contentment with their son’s medical care was similar (DMD 79%; BMD 82%) whereas only 0.5% (DMD) and 3% (BMD) parents claimed to be very unsatisfied (data not shown).

### Estimation of total disease burden

Regarding all analyzed influencing factors of direct and indirect costs from different perspectives of patients, caregivers and families, considerably higher mean costs were identified for DMD (p < 0.001). Thus, direct COI of DMD (€ 50,230) was nearly three times higher than in BMD (€ 17,611) showing the highest COI in the most severe clinical stage (Tables 4 and 5). Indirect costs were approximately 1.3 times higher for DMD (€ 28,683) when compared to BMD (€ 21,449). In total, the mean economic burden of DMD per year was estimated at € 78,913 versus € 39,060 in BMD. Total health care burden was significantly higher in DMD than in BMD (p < 0.001). Recently, the worldwide prevalence of DMD was described as 4.78:100,000 males in DMD, and 1.53:100,000 males in BMD [15]. Recent calculations of the German population [16] estimated that about 1,900 DMD patients and 600 BMD patients may live in Germany. Altogether, this implicates the annual disease burden of DMD to be € 150 million which is more than six times higher than the BMD health care burden with an estimated € 23 million.

## Discussion

Our study provides the first German analysis of the DMD health care burden which not only assesses the impact of disease severity and progression on disease burden but also compares the economic burden to the clinically milder allelic BMD disease phenotype. To date, we assessed the largest German patient cohort suffering from DMD and BMD, analyzing different cost driving factors from diverse perspectives. All enrolled patients had a genetically confirmed diagnosis of DMD or BMD resulting in a homogenous patient cohort. Total estimated disease burden was significantly higher for DMD (€ 78,913) when compared to BMD (€ 39,060; p < 0.001). The total COI showed a clear increase with disease severity as similarly reported for different neuromuscular disorders before [17,18]. The nation-wide annual total DMD burden of illness in Germany was 6.5 times higher than the estimated BMD burden (€ 150 vs. 23 million).

An estimation of annual per-patient direct COI showed nearly four times higher costs of DMD than of BMD, resulting from a higher demand of direct medical care, nursing services and non-medical resources. Major identified cost drivers of direct costs were informal care costs (DMD 27%/BMD 20% of COI), costs of medical aids (DMD 13%/BMD 2% of COI) and costs of rehabilitation services (DMD/BMD 6% of COI) along with costs for the housing situation (DMD 5%/BMD 6% of COI). These results correspond with a recent study on facio-scapulo-humeral muscular dystrophy (FSHD) emphasizing the high impact of mobility impairment, activity limitation and impairment of social roles on disease burden [19]. Thus, devices that help to maintain these abilities and allow for continued social participation play an important role, and might even outbalance the importance of direct medical treatment as shown by our cost estimate. Interestingly, non-medical costs such as travel expenses (e.g. due to medical consultations and treatment) and costs for personal assistance in DMD were higher than the mean medical costs of drug treatment or outpatient medical consultations. Comparing our study results to a recently published international cross-sectional study on DMD disease burden [8], we estimated higher direct costs (€ 50,230 vs. US $42,360) probably resulting from a different methodology of cost estimation. There are several discrepancies comparing our results to those of previous studies, since different national health care systems or different types of muscular dystrophies with inhomogeneous patient cohorts were analyzed [20,21], which do not allow a close comparison.

Informal care costs were identified as a main cost driving factor of disease burden in both DMD and BMD, with increasing expenses along with disease progression (mean informal COI € 21,279 vs. 7,636). In DMD, we found a small cost reduction from stage I to II, most likely because the very young DMD patients in stage I (median age 4y) as toddlers require full attendance of a caregiver despite a mild clinical phenotype. The high standard deviations for care time display the wide range of responses (0.5 – 24 hours per day) and possibly hint at the patients’ and parents’ difficulties to estimate care efforts. However, 66% of caring parents were working and therefore our results may underestimate the actual amount of informal care costs, since working parents have been excluded from our calculation to prevent the double counting of indirect and informal care costs. If the care efforts of working parents would have been incorporated, the informal care costs would have been much higher. This highlights the enormous loss of working capacity and productivity due to informal care required for dystrophinopathies, and in particular for DMD. Recently published data confirmed these findings by showing that indirect costs of neuromuscular diseases - calculated as a loss of family income - are directly influenced by the level of informal care needed by the patients [21]. In contrast, another recent analysis showed multiple times higher indirect costs compared to ours by using the human capital approach [8], whereas we calculated indirect costs with the actual wage of patients and parents. Therefore, our results might be more precise and lower.

However, we did not only assess the reduced productivity, but additionally analyzed parents’ medical problems obtaining further information on the duration and cause of absenteeism from work. Parental medical problems based on their son’s disease and related care were twice as frequent in DMD than in BMD. Orthopedic and psychiatric disorders were quite frequent with a higher ratio of psychiatric disorders in BMD parents than in DMD parents. In contrast, more than half of the DMD parents complained of sleep disorders compared to 31% of the BMD parents, which may hint at still unrecognized psychiatric diseases in DMD parents. Nevertheless the age-matched prevalence of these medical problems within the general population still has to be analyzed before we can assume a causal relation between the parents’ medical problems and their son’s disease burden. However, the parents’ own medical problems increased along with the disease progression of their son, which is in line with the correlation between patients’ disease burden and the general disease severity.

Furthermore, we analyzed indirect costs due to patients’ own loss of productivity - an enormous cost factor which has not been previously analyzed for DMD and BMD patients - which was three times higher than indirect costs caused by parents and caregivers in DMD patients, and even 7.5 times higher in BMD. Thus, our results show the high relevance of indirect costs: in DMD, indirect costs caused 36% of total burden of illness, in BMD, the percentage was even higher (55% of total COI) due to the higher education and employment rate reflecting a slower disease progression and resulting in a higher gross salary per year compared to DMD patients. We assumed that working life starts at age 20 years and calculated a potential salary for each non-working DMD and BMD patient over 20 years. Due to the low median age of DMD patients (11 y), 86% of DMD patients did not cause any indirect costs. On the contrary, indirect costs caused by parents were notably higher for DMD than for BMD because of the reduced autonomy of DMD patients. Real-life parents’ indirect costs may be even higher than we estimated since only the productivity loss of one caregiver was taken into account. Differences between patients’ and parents’ indirect costs can be explained by our methodology. To prevent an overestimation of parents’ loss of productivity due to quitting work, we only included parents who stopped working in 2013 whereas for patients all lost working days even before 2013 were included, assuming that their employment status was directly illness-related. Compared to the general German population, mean gross salaries of BMD/DMD patients and parents were lower (mean of total German population € 41.388 in 2013; [22]) and the percentage of non-working patients and parents was notably higher (mean of total German population 5.2%; [23]), illustrating the limitations in working life of both DMD and BMD patients and parents.

A possible underestimation of the burden of illness by using minimum prices may be a weakness of our study, since it is not possible to identify the exact incurred costs without using data from health insurances. Additionally, patients and parents were asked to complete questions concerning the consumption of resources for a defined period in the past, whereas recall bias may have led to errors. Another limitation of our study may be a possible selection bias by patient recruitment with help of the German DMD/BMD patient registry, since patients and families voluntarily participating in the DMD patient registry may represent the more compliant and motivated patient cohort in general. The main aim of the DMD patient registry is to facilitate the planning of clinical trials and to enhance patient recruitment into these trials; therefore, older patients in more severe clinical stages may be underrepresented since they do not represent the target group usually participating in clinical trials, which may also lead to cost underestimation. Finally, since BMD displays a more variable clinical course and a milder phenotype than DMD, patients may face difficulties in specifying their clinical severity stage which may result in a lower mean age in stage III compared to stage II.

Regarding patients’ self-rated HRQOL, we identified a decrease of quality of live with increasing clinical severity. Surprisingly, DMD patients assessed their own HRQOL worse than their parents did. Recently, differences in self- and parent-reported HRQOL in DMD were described mainly in non-observable dimensions like worries/evaluation of familial problems and communication [24]. In contrast, BMD’s HRQOL was similarly reported by patients and parents, possibly due to the more mature age and thereby advanced ability to talk about worries and impairments. An advantage of our study is the use of a disease-specific measurement tool and the assessment of both patients’ and parents’ perspectives, which gives a more precise picture of HRQOL in dystrophinopathy patients.

Overall, our analysis of the DMD and BMD health care burden identified indirect costs, informal care costs and costs of medical aids and rehabilitation services as the most important cost drivers. In DMD, the socio-economic burden is manifold higher than in BMD, increasing with disease progression and severity in both DMD and BMD, thus leading to a similar burden of DMD and BMD in the highest clinical severity stages.

Novel curative therapeutic strategies are currently developed which aim at the correction of the genetic defect and thereby restoring muscle protein function to slow down disease progression or to result in a milder clinical phenotype [25]. Pharmaceutical and clinical development of these promising therapies are costly and once approved they may add eminent costs to health care budgets. Nevertheless, our results suggest that stopping DMD and BMD disease progression at an early stage or modifying the severity of DMD into a milder BMD-like phenotype is likely to reduce total disease burden along with considerably lower costs and improvement of patients’ quality of life.

## Conclusion

In conclusion, early assessments of economic aspects and total disease burden represent the first analytical steps towards a systematic health economic assessment of dystrophinopathies in the light of rising innovative therapeutic approaches. In this context, our study adds to the validity of estimation with its comprehensive approach and its execution within a normal care environment. With emerging therapeutic strategies aiming at dystrophin restoration and improved muscular function, along with a milder clinical phenotype, our results show that it is not only necessary for the patients, but also economically justified to explore new therapies that modify the clinical severity or slow down the progression of the disease. This would lower the total economic disease burden to a high extent and improve patients’ HRQOL. Thus, our results may speed up payer negotiations regarding pricing and reimbursement and contribute to the facilitation of an efficient translation of innovations from clinical research over marketing authorization to patient access to a reimbursed therapy.

## Additional file

Additional file 1:
**Formula for calculation of indirect costs.**

## References

[1] Mendell JR, Lloyd-Puryear M (2013). Report of MDA muscle disease symposium on newborn screening for Duchenne muscular dystrophy. Muscle Nerve.

[2] Moat SJ, Bradley DM, Salmon R, Clarke A, Hartley L (2013). Newborn bloodspot screening for Duchenne muscular dystrophy: 21 years experience in Wales (UK). Eur J Hum Genet.

[3] Bushby K, Finkel R, Birnkrant DJ, Case LE, Clemens PR, Cripe L, Kaul A, Kinnett K, McDonald C, Pandya S, Poysky J, Shapiro F, Tomezsko J, Constantin C, D. M. D. Care Considerations Working Group: **Diagnosis and management of Duchenne muscular dystrophy, part 1: diagnosis, and pharmacological and psychosocial management.** *Lancet Neurol* 2010, **9:**77-93. 10.1016/S1474-4422(09)70271-6 19945913

[4] Bushby K, Finkel R, Birnkrant DJ, Case LE, Clemens PR, Cripe L, Kaul A, Kinnett K, McDonald C, Pandya S, Poysky J, Shapiro F, Tomezsko J, Constantin C, D. M. D. Care Considerations Working Group: **Diagnosis and management of Duchenne muscular dystrophy, part 2: implementation of multidisciplinary care.** *Lancet Neurol* 2010, **9:**177-189. 10.1016/S1474-4422(09)70272-8 19945914

[5] Eagle M, Baudouin SV, Chandler C, Giddings DR, Bullock R, Bushby K (2002). Survival in Duchenne muscular dystrophy: improvements in life expectancy since 1967 and the impact of home nocturnal ventilation. Neuromuscul Disord.

[6] Bushby KM, Thambyayah M, Gardner-Medwin D (1991). Prevalence and incidence of Becker muscular dystrophy. Lancet.

[7] Bushby KM, Gardner-Medwin D (1993). The clinical, genetic and dystrophin characteristics of Becker muscular dystrophy. I. Natural history. J Neurol.

[8] Landfeldt E, Lindgren P, Bell CF, Schmitt C, Guglieri M, Straub V, Lochmuller H, Bushby K (2014). The burden of Duchenne muscular dystrophy: An international, cross-sectional study. Neurology.

[9] Bladen CL, Rafferty K, Straub V, Monges S, Moresco A, Dawkins H, Roy A, Chamova T, Guergueltcheva V, Korngut L, Campbell C, Dai Y, Barisic N, Kos T, Brabec P, Rahbek J, Lahdetie J, Tuffery-Giraud S, Claustres M, Leturcq F, Ben Yaou R, Walter MC, Schreiber O, Karcagi V, Herczegfalvi A, Viswanathan V, Bayat F, de la Caridad Guerrero Sarmiento I, Ambrosini A, Ceradini F *et al*: **The TREAT-NMD Duchenne muscular dystrophy registries: conception, design, and utilization by industry and academia.** *Hum Mutat* 2013, **34:**1449-1457. 10.1002/humu.22390 23913485

[10] Lauer-Fischer GmbH: **Die LAUER-Taxe 2013.** Fürth, Germany: LAUER-FISCHER GmbH; 2013.

[11] **Verband der Ersatzkassen: Rahmenverträge und Vergütungslisten bei Heilmitteln** [https://www.vdek.com]

[12] Krauth C, Hessel F, Hansmeier T, Wasem J, Seitz R, Schweikert B (2005). [Empirical standard costs for health economic evaluation in Germany – a proposal by the working group methods in health economic evaluation]. Gesundheitswesen.

[13] Statistisches Bundesamt: Sozialleistungen - Angaben zur Krankenversicherung. Statistisches Bundesamt: Wiesbaden; 2012.

[14] **Bundesministerium für Gesundheit (BMG): Pflegeversicherung** [http://www.bmg.bund.de/pflege/pflegeversicherung.html]

[15] Mah JK, Korngut L, Dykeman J, Day L, Pringsheim T, Jette N (2014). A systematic review and meta-analysis on the epidemiology of Duchenne and Becker muscular dystrophy. Neuromuscul Disord.

[16] **Statistisches Bundesamt: Bevölkerung auf Grundlage des Zensus 2011** [https://www.destatis.de/DE/ZahlenFakten/GesellschaftStaat/Bevoelkerung/Bevoelkerungsstand/Tabellen/Zensus_Geschlecht_Staatsangehoerigkeit.html]

[17] Schepelmann K, Winter Y, Spottke AE, Claus D, Grothe C, Schroder R, Heuss D, Vielhaber S, Mylius V, Kiefer R, Schrank B, Oertel WH, Dodel R: **Socioeconomic burden of amyotrophic lateral sclerosis, myasthenia gravis and facioscapulohumeral muscular dystrophy.** *J Neurol* 2010, **257:**15-23. 10.1007/s00415-009-5256-6 19629566

[18] Kanters TA, Hagemans ML, van der Beek NA, Rutten FF, van der Ploeg AT, Hakkaart L (2011). Burden of illness of Pompe disease in patients only receiving supportive care. J Inherit Metab Dis.

[19] Johnson NE, Quinn C, Eastwood E, Tawil R, Heatwole CR (2012). Patient-identified disease burden in facioscapulohumeral muscular dystrophy. Muscle Nerve.

[20] Ouyang L, Grosse SD, Kenneson A (2008). Health care utilization and expenditures for children and young adults with muscular dystrophy in a privately insured population. J Child Neurol.

[21] Larkindale J, Yang W, Hogan PF, Simon CJ, Zhang Y, Jain A, Habeeb-Louks EM, Kennedy A, Cwik VA (2014). Cost of illness for neuromuscular diseases in the United States. Muscle Nerve.

[22] **Statistisches Bundesamt: Entwicklung der Bruttoverdienste.** [https://www.destatis.de/DE/ZahlenFakten/GesamtwirtschaftUmwelt/VerdiensteArbeitskosten/VerdiensteVerdienstunterschiede/Tabellen/LangeReiheD.html]

[23] **Statistisches Bundesamt: Volkswirtschaftliche Gesamtrechnungen** [https://www.destatis.de/DE/ZahlenFakten/GesamtwirtschaftUmwelt/Arbeitsmarkt/Erwerbslosigkeit/Tabellen/EinwohnerErwerbsbeteiligung.html]

[24] Uzark K, King E, Cripe L, Spicer R, Sage J, Kinnett K, Wong B, Pratt J, Varni JW (2012). Health-related quality of life in children and adolescents with Duchenne muscular dystrophy. Pediatrics.

[25] Aartsma-Rus A, Van Ommen GJ, Kaplan JC (2013). Innovating therapies for muscle diseases. Handb Clin Neurol.

