# Supplementary material for: Comparative cost of illness analysis and assessment of health care burden of Duchenne and Becker muscular dystrophies in Germany
Source: Orphanet J Rare Dis. 2014 Dec 18;9:210. doi: 10.1186/s13023-014-0210-9 (PMC4302713; doi:10.1186/s13023-014-0210-9)
Supplement: Additional file 1: — Formula for calculation of indirect costs. [file 13023_2014_210_MOESM1_ESM.pdf]

Additional file:

Formula for calculation of indirect costs caused by patients:

$$Loss\ of\ productivity\ (2013) = LP_{disability} + LP_{invalidity}$$

Loss of productivity due to disability: $LP_{disability} = (Abs_{LT} + Abs_{ST} + Rehab) \times S_d + S_{lost} \times t$

Abs_{LT}: long-term absenteeism due to BMD/DMD in one year (in days)
Abs_{ST}: short-term absenteeism - absent hours due to BMD/DMD in one year and conversion into days
Rehab: days in rehabilitation in one year
S_d: actual gross salary per day
S_{lost}: loss of gross salary per month caused by changes in working hours
t: number of months with reduced salary due to changes in working hours in 2013

Loss of productivity due to invalidity: $LP_{inval} = \mathrm{D}_{\mathrm{lost}} \times S_{di}$

D_{lost}: lost working days due to DMD/BMD in one year
S_{di}: previous gross salary per day, inflation adjusted
Patients who never worked: average gross salary per month for men in Germany in 2013 [1] extrapolated to one year

Formula for calculation of indirect costs caused by parents:

$$Loss\ of\ productivity\ (2013) = LP_{absenteeism} + LP_{changes}$$

Loss of productivity due to absenteeism: $LP_{absenteeism} = (Abs_{LT} + Abs_{ST} + Rehab) \times S_d$

Abs_{LT}: long-term absenteeism due to child's BMD/DMD in one year (in days)
Abs_{ST}: short-term absenteeism - absent hours due to child's BMD/DMD in one year and conversion into days
Rehab: days in rehabilitation in one year
S_d: actual gross salary per day

Loss of productivity due to changes in work situation: $LP_{change} = S_{lost} \times t + \mathrm{D}_{\mathrm{lost}} \times S_{di}$

S_{lost}: loss of gross salary per month caused by changes in working hours due to child's disease
t: number of months with reduced salary due to changes in working hours in 2013
D_{lost}: lost working days due to child's DMD/BMD in 2013
S_{di}: previous gross salary per day, inflation adjusted

References:

1. **Statistisches Bundesamt. Verdienste und Arbeitskosten. Arbeitnehmerverdienste.** vol. Fachserie 16, Reihe 2.3. Wiesbaden; 2014.
